# Circadian patterns of growth factor receptor-dependent signaling and implications for carcinogenesis

**DOI:** 10.1186/s12964-024-01676-w

**Published:** 2024-06-10

**Authors:** Emanuele Murgo, Giorgia Falco, Gaetano Serviddio, Gianluigi Mazzoccoli, Tommaso Colangelo

**Affiliations:** 1 Department of Medical Sciences, Division of Internal Medicine and Chronobiology Laboratory, Fondazione IRCCS “Casa Sollievo della Sofferenza”,, Opera di Padre Pio da Pietrelcina, San Giovanni Rotondo, 71013 Italy; 2 Department of Medical and Surgical Sciences, University of Foggia, Foggia, Italy; 3 Cancer Cell Signaling Unit, Fondazione IRCCS “Casa Sollievo della Sofferenza”, San Giovanni Rotondo (FG), San Giovanni Rotondo, Italy

**Keywords:** Growth factors, Receptors, Ligand, Circadian, Biological clock, Cancer

## Abstract

Several different signaling pathways that regulate cell proliferation and differentiation are initiated by binding of ligands to cell-surface and membrane-bound enzyme-linked receptors, such as receptor tyrosine kinases and serine-threonine kinases. They prompt phosphorylation of tyrosine and serine-threonine residues and initiate downstream signaling pathways and priming of intracellular molecules that convey the signal in the cytoplasm and nucleus, with transcriptional activation of specific genes enriching cell growth and survival-related cascades. These cell processes are rhythmically driven by molecular clockworks endowed in every cell type and when deregulated play a crucial role in cancer onset and progression. Growth factors and their matching receptor-dependent signaling are frequently overexpressed and/or dysregulated in many cancer types. In this review we focus on the interplay between biological clocks and Growth Factor Receptor-dependent signaling in the context of carcinogenesis.

## Introduction

In humans, growth factor receptors (GFRs) superfamily includes proteins capable of transduce extracellular signals to cellular responses and in particular to prompt mitogenic activity of growth factor ligands. Heterogeneous molecular signals are conveyed across the cytoplasm to the transcriptional machinery into the nucleus through sequential kinase signaling, settled in redundant and cross-talking cascades [1, 2]. GFRs are sited in the cell plasma membrane as monomers or (pre)dimers and ligand binding elicits higher-order oligomerization of ligand-receptor complexes. GFRs encompass three domains: (i) extracellular ligand (growth factor) binding domain, (ii) transmembrane domain, (iii) cytoplasmic domain with enzymatic activity or complexing with enzymatic proteins [1, 2]. The expression of genes encoding GFRs is positively or negatively regulated at the transcriptional level through protein-DNA and protein–protein interactions in various cell types, depending on the developmental and cellular context. Establishment of the ligand-receptor complex through interaction of the three-dimensional structure of ligands with the related members of the GFR tyrosine kinase (RTK) or serine-threonine kinase (RSTK) superfamily initiates the signal transduction cascade via auto-phosphorylation by the intracellular tyrosine or serine-threonine kinase domain [1, 2]. The organization of the extracellular portion of the receptor enclosing the ligand binding domain differs considerably among the different superfamily members and binding of growth factors to their cell plasma membrane receptors elicits phosphorylation of tyrosine or serine-threonine residues on numerous intracellular signaling molecules, which transmit the signal in the cytoplasm and nucleus [1, 2]. Mainly, GFRs signaling through RTKs actuate molecular cascades such as phosphatidylinositol 3-kinase (PI3K) pathway, mitogen-activated protein kinase (MAPK) via the Rat sarcoma/rapidly accelerated fibrosarcoma/mitogen-activated protein kinase (RAF-MKK1/2-Erk1/2/MAPK kinase cascade) and JAK2/STAT signaling. RSTKs encompass a set of related catalytic receptors comprising TGF-β receptors (TGF-βRI-III) and activin receptors (ALK1-7) existing as heterodimers of type I and type II receptors, with the ligand binding domain located in the type II receptor [1, 2]. Upon ligand-receptor binding, the type I receptors are recruited to the complex and are phosphorylated by the type II receptor [1, 2]. Activated type I receptors subsequently phosphorylate SMAD proteins, depending in detail on the RSTK subtype: TGF-βR and ALK4/5/7 phosphorylate SMAD2/3, whereas ALK1/2/3/6 phosphorylate SMAD1/5/8. Upon activation, SMAD proteins complex with SMAD4 and after conveyance into the nucleus bind transcription factors and co-factors to control expression of target genes [1, 2]. A wide-ranging pathway atlas for EGFR-mediated signaling comprises EGFR endocytosis with sequential degradation or recycling, small guanosine triphosphatase (GTPase)-mediated signal transduction, MAPK and PI3K signaling, and G protein-coupled receptor (GPCR)-mediated EGFR transactivation through intracellular Ca^2+^ signaling [1, 2]. Deranged intracellular signaling plays a crucial role in oncogenesis and is a target of therapeutic interventions [3–6]. The expression of genes encoding GFRs and their specific ligands oscillates rhythmically and show patterns defined circadian (from the Latin words *circa* and *dies*, approximately 24 h) [7]. Circadian rhythms of biological processes are driven by the circadian clock circuitry, a hierarchical organization of molecular clockworks ticking in every cell of the body and working through a molecularly hardwired negative feedback loop with temporal delay [8].

### The circadian clock circuitry

On our planet, life forms adjusted to 24-hour light/darkness and temperature transition and seasonal rhythmicity, related to Earth’s spin on its axis and revolution motion around the Sun, respectively, developing endogenous molecular clockworks to synchronize their behavioral and metabolic rhythms to predictable environmental cues [9, 10]. The harmonized regulation of sleep/wake, rest/activity, fasting/feeding cycles with biochemical processes and metabolic pathways is crucial to maintain body homeostasis and preserve health. Coordinating biological rhythms and anticipated changes of spatio/temporal niches impacts organism and species survival of free-living animals, influencing behavioral cycles of feeding, predation, competition, mating and providing fitness advantage as opposed to natural selection pressure [11–13]. Loss of resonance of body rhythmicity with external synchronizers or a change of oscillation phase may alter the physiological array of rhythms with onset of internal desynchronization, also known as chronodisruption, which promotes neoplastic, metabolic, inflammatory and neurodegenerative diseases [11–13]. Daily timekeeping is driven in mammals and humans by the circadian timing system, comprising a central pace-maker and master oscillators located in the suprachiasmatic nuclei (SCN) of the hypothalamus and autonomous self-sustained oscillators in every cell of peripheral tissues. Ambient natural or artificial photic cues are perceived by non-image forming intrinsically photosensitive retinal ganglion cells containing melanopsin, whose output is transferred to the SCN by the retino-hypothalamic tract [14–16]. SCN coordinate brain nuclei and peripheral oscillators through neural and hormonal signals, represented by autonomic nervous system innervation and circulating systemic factors (cortisol during the day and melatonin at night) [17]. The biological oscillators in every cell are driven by molecular clockworks ticking through a set of genes whose expression oscillates with circadian rhythmicity, as well as the encoded proteins that in turn block their transcriptional activation, operating a loop in which the product of gene transcription in sequence and with delay decreases gene expression [14–16]. Peripheral oscillators are entrained also by food assumption and fluctuation of nutrient levels, while central oscillators in the SCN are resilient to feeding-related stimuli and are entrained primarily by light/darkness alternation, so that desynchronization of food intake and metabolic processes respect to proper diurnal activity patterns uncouple the light-driven SCN and tissue/organ systems oscillators [14–16].

### The molecular clockwork

**Fig. 1:**
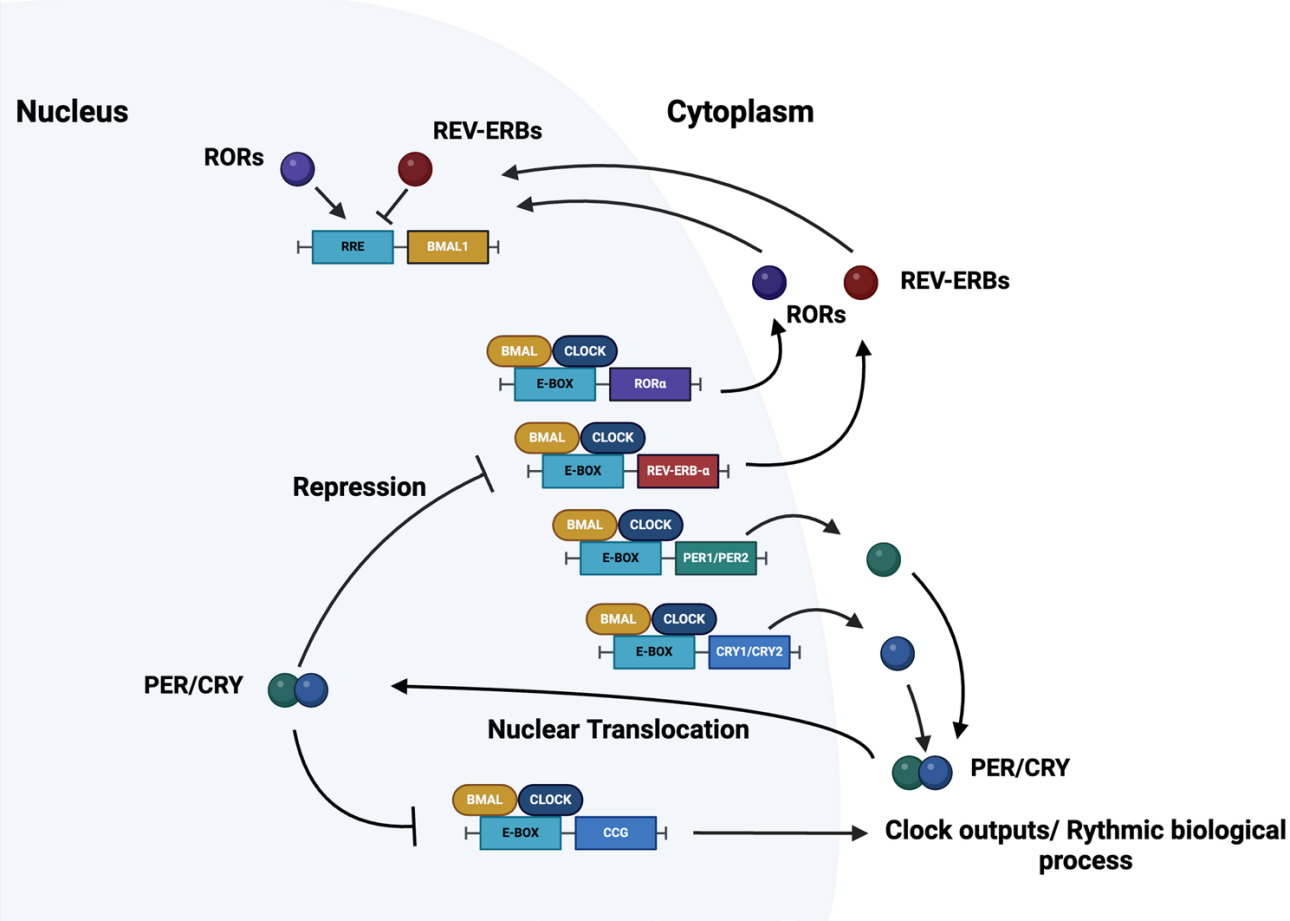
Outline of the molecular clockwork. The biological clock is hard-wired by interlocking transcriptional–translational feedback loops (TTFLs) operated by a set of circadian protein-encoding genes. The positive limb of the TTFL is ran by the transcription factors BMAL1 or its analog BMAL2 (schematically rendered as BMAL) and CLOCK, which heterodimerize and bind to E-box enhancer elements in the promoters of the Period genes (PER1-2) and Cryptochrome genes (CRY1-2). PER and CRY proteins operate the negative limb of the TTFL and hinder the transcriptional activity of BMAL:CLOCK heterodimers. The nuclear receptors REV-ERBs and RORs compete at specific response elements (RRE) on the promoter of BMAL1 and drive its rhythmic expression. See text for in depth explanation and details

The molecular cogs operating to drive circadian rhythmicity at the cellular level are represented by a group of genes, named core clock genes, and their encoded.

proteins that manage interlocking transcription-translation feedback loops (TTFLs), in addition to non-transcriptional loops, carrying out one cycle in roughly 24 h [18–21]. The TTFL is worked by a positive limb, operated by the bHLH-PAS (basic helix-loop-helix–Period-Arnt-Single-minded) transcriptional activators CLOCK (circadian locomotor output cycles kaput), and its paralog NPAS2 (neuronal PAS domain protein 2), and BMAL1-2/ARNTL-2 (brain and muscle aryl-hydrocarbon receptor nuclear translocator-like/aryl-hydrocarbon receptor nuclear translocator-like) that heterodimerize and bind to enhancer (E)-box DNA consensus sequences of the target Period (*PER1-3*) and Cryptochrome (*CRY 1–2*) genes [18–21]. The encoded PER1-3 and CRY1-2 proteins operate the TTFL negative limb; they accrue and dimerize in the cytoplasm forming repressor complexes that pass back into the nucleus and inhibit the transcriptional activity of CLOCK: BMAL1-2 heterodimers [18–21]. The circadian proteins undergo post-translational modifications (PTMs), such as phosphorylation, acetylation, sumoylation, O-GlcNAcylation) modulating their activity and in sequence ubiquitination/proteasomal degradation allowing correct functioning of the TTFL and setting of biological clock speed [22–24]. An auxiliary interconnected loop is operated by the nuclear receptors (NRs) REV-ERBs and retinoic acid-related (RAR) orphan receptor (RORs), which drive BMAL1 rhythmic transcription competing for a ROR specific response elements (RORE) in its promoter [25]. ROR-α works as transcription activator and physically interacts with peroxisome proliferator-activated receptor (PPAR)-γ coactivator-1α (PGC-1α), which recruits chromatin-remodelling complexes to proximal *BMAL1* promoters and elicits *BMAL1* transcription. Conversely, REV-ERB-α interacts with the nuclear corepressor/histone deacetylase3 (NCoR-HDAC3) corepressor complex and inhibits BMAL1 transcription [25]. Other than the NR operated feedback loop, CLOCK: BMAL1 heterodimers through cognate D-box elements regulate the expression of the PAR domain basic leucine zipper (bZIP) transcription factors and first order clock controlled gens DBP (albumin D-site binding protein), TEF (thyrotroph embryonic factor), HLF (hepatic leukaemia factor), which successively drive the rhythmic expression of thousands tissue specific (output) genes [26, 27]. Besides, REV-ERBs and DBP competing for Res drive the expression of the Nuclear factor, interleukin 3 regulated protein (NFIL3, also known as E4BP4), whose promoter contains a RORE, so that its transcription is suppressed by REV-ERBs and shows an oscillatory pattern with opposite phase respect to *DBP* [26, 27]. Other circadian components involved in the molecular clockwork are the E-box-binding basic helix-loop-helix transcription factors DEC1 (Differentially expressed in chondrocytes protein 1) and DEC2 [26, 27]. In particular, *DEC1* transcription is elicited by CLOCK: BMAL1 heterodimer, but in turn DEC1 proteins repress CLOCK: BMAL1 transcriptional activity, operating a power steering feed-back [26, 27]. Epigenetic modifications fine tune the functioning of the molecular clockwork through rhythmic chromatin-histone remodelling and are mainly primed by acetylation/deacetylation as well as methylation/demethylation processes. Regarding the cogs of the molecular clockwork, BMAL1 acetylation is operated by CLOCK, which has intrinsic protein and histone acetyltransferase capability [28]. SIRT1, a type III histone/protein deacetylase, counters this process and its activity depends on the intracellular levels of nicotinamide adenine dinucleotide (NAD+), a nutrient sensor synthesized from tryptophan through the enzymatic activity of nicotinamide phosphoribosyl-transferase (NAMPT/visfatin), whose expression is rhythmically driven by the biological clock [29–32]. (Fig. 1).

### Epidermal growth factor receptor-dependent signaling

In mammalian cells the Epidermal Growth Factor Receptor (EGFR) signaling pathway controls essential functions such as survival, proliferation and migration. Different subtypes of erythroblastic leukemia viral (v-erb-b) oncogene homolog (ErbB) receptors bind a family of ligands with activation of various molecules in a wide-ranging system of receptor complexes expressed on near every human cell type [33]. Ligand binding prompts homo- and hetero-dimerization of four ErbB family receptors: ErbB1 (also known as EGFR), ErbB2, ErbB3, and ErbB4 [2]. EGFR ligands comprise several molecules, among which transforming growth factor (TGF)-α, amphiregulin, heparin-binding EGF-like growth factor (HB-EGF) and epiregulin, decisively entailed in tissue-specific proliferation/differentiation homeostasis. Binding of ErbB dimer by a definite ligand elicits ErbB cytoplasmic kinase activity and starts auto- and transphosphorylation on tyrosine residues, providing a docking site for adaptor proteins and enzymes, while the ATP binding pocket is held between the two lobes of the kinase fold [2]. EGFR activation may occur with numerous mechanisms under physiological or pathological conditions. In addition to direct activation by specific ligands, heterologous ligand-dependent mechanisms are likewise involved, such as EGFR activation through G-protein-coupled receptors (GPCRs) activation by mature forms of EGFR ligands cleaved by metalloproteinase from membrane precursors [2].

EGFR ligand-independent mechanisms may be also involved. For instance, integrins may form physical complexes with GFRs at the cell plasma membrane. Also, transactivation from GPCR cytokine receptors may play a role, regulating pro-inflammatory activation and transducing modifications in proliferation rate as well as cellular shape and attachment [2].

**Fig. 2:**
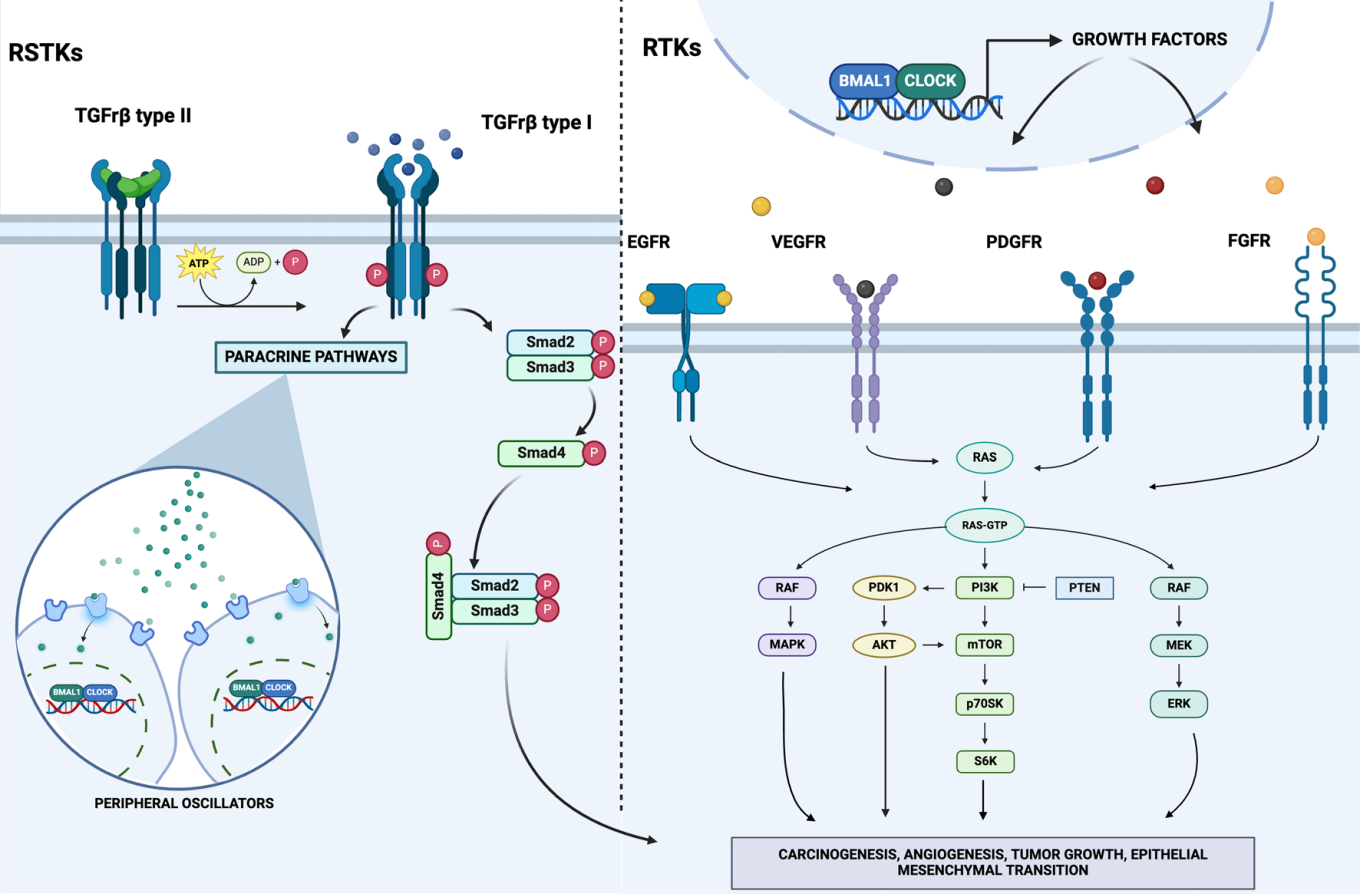
Schematic representation of intracellular signaling prompted by Receptor Tyrosine Kinases (RTKs) and Receptor Serine/Threonine Kinases (RSTKs). See text for in depth explanation and details

Conversely, EGFR functions can be constrained through de-phosphorylation by active protein-tyrosine phosphatases, which sequentially may be reversibly inactivated through oxidation of the catalytic cysteine residue in their active site by reactive oxygen species produced upon EGFR activation [2]. The EGF family mediators bind with autocrine/paracrine pattern their receptors through the ligand binding site on the extracellular domain of cell plasma membrane receptors and elicit an active reparative response in case of biophysical damage, but can also play a role in deranged cell processes involved in carcinogenesis. Ligand binding prompts homo- and hetero-dimerization of the receptor inducing intracellular RTK activation. On their side, the IGF-1 and insulin receptors exist as covalent cross-linked dimers with each monomer comprising two subunits [2]. Growth factor signaling can be terminated by receptor-mediated endocytosis with internalization of the ligand-receptor complex, but in case of oncogenic changes RTKs signal unrelenting cell proliferation regardless of growth factor stimulation [34]. In human cancers constitutive RTK activation is initiated by gain-of-function mutations, genomic amplification, chromosomal rearrangements, and autocrine activation. Mutated forms of growth factor proteins with derangement of GFR activation hallmark virtually all epithelial cancers, but on the other hand represent valuable targets for therapeutic agents [34].(Fig. 2; Table 1).

**Table 1:** List of receptor tyrosine kinases

*ALK/CD246*	*Anaplastic Lymphoma Kinase (ALK), also known as CD246, is a 220 kDa transmembrane glycoprotein that is primarily expressed in the developing nervous system.*
*Axl, Dtk and Mer*	*Member of the TAM family with the high-affinity for the ligand growth arrest-specific protein 6 (GAS6), a vitamin K-dependent protein that is structurally related to the anticoagulation factor protein S.*
*DDR1/ DDR2*	*Discoidin domain receptor 1 (DDR1) is a receptor tyrosine kinase that binds and is activated by collagens*
**EGFR/ HER1-6/ ErbB-1-6**	Transmembrane protein that is a receptor for members of the epidermal growth factor family
*Eph receptor (A1-10;B1-4)*	*Tyrosine kinases receptors that bind membrane-bound ephrin ligands, which are expressed on neighboring cells. Eph receptor?ephrin complexes emanate bidirectional signals that affect both receptor- and ephrin-expressing cells*
**FGF R1-4**	**Receptors that bind to members of the fibroblast growth factor (FGF) family of proteins that produce mitogenic and angiogenic effects in target cells by signaling through cell surface receptor tyrosine kinases**
*Flt-3/Flk*	*Receptor tyrosine kinase expressed by immature hematopoietic progenitor cells. The ligand for FLT3 is a transmembrane or soluble protein and is expressed by a variety of cells including hematopoietic and marrow stromal cells*
*HGF R/c-MET*	*Receptor tyrosine kinase that, after binding with its ligand, hepatocyte growth factor, activates a wide range of different cellular signaling pathways, including those involved in proliferation, motility, migration and invasion.*
*IGF-I R*	*Disulfide-linked heterotetrameric transmembrane protein consisting of two alpha and two beta subunits that binds insulin-like growth factor with a high affinity.*
*Insulin R/CD220*	*Type I transmembrane single chain preproprotein with a putative 27 amino acid residues (aa) signal peptide*
*M-CSF R*	*Member of the type III subfamily of receptor tyrosine kinases, Macrophage Colony-Stimulating Factor Receptor (M-CSF R), also known as Colony Stimulating Factor 1 Receptor (CSF1 R) and CD115, is a cell-surface protein that serves as a receptor for the cytokines M-CSF and IL-34.*
*MSP R/Ron*	*Macrophage stimulating protein receptor (MSPR), encoded by the human RON genes is synthesized as a single-chain precursor that is cleaved into a mature disulfide-linked heterodimer composed of an extracellular ? chain and a membrane spanning ? chain with intrinsic tyrosine kinase activity.*
*MuSK*	*Muscle-Specific Kinase is a receptor tyrosine kinase required for the formation and maintenance of the neuromuscular junction.*
**PDGF R ?** / **PDGF R ?**	Cell surface tyrosine kinase receptor for members of the platelet-derived growth factor family. These growth factors are mitogens for cells of mesenchymal origin. PDGFR? (CD140a) and PDGFR?(CD140b) activate many overlapping signaling pathways
*RET*	*Rearranged during transfection (RET) is a tyrosine kinase receptor that under normal circumstances interacts with ligand at the cell surface and mediates various essential roles in a variety of cellular processes such as proliferation, differentiation, survival, migration, and metabolism. Mutations either activating or inhibiting RET result in several aggressive cancers*
*ROR1*	*Receptor tyrosine kinase like orphan receptor 1 is a receptor tyrosine kinase-like orphan receptor that modulates neurite growth in the central nervous system.*
*ROR2*	*Functions as a noncanonical Wnt receptor that regulates NMDAR-mediated synaptic transmission.*
*Ryk*	*Receptor like tyrosine kinase, is a receptor of noncanonical Wnt ligand Wnt5a and is positively correlated with gastric cancer tumorigenesis and potential of liver metastasis*
*SCF R/c-kit*	*The c-kit receptor (CD117) is a transmembrane protein with tyrosine kinase activity encoded by the oncogene c-kit. It is an important member of type III receptor tyrosine kinase family. The ligand for c-kit is stem cell factor (SCF), a hematopoietic cytokine, which plays an important role in maintaining the survival of hematopoietic cells*
*Tie-1/Tie-2*	*Tie1 and Tie2 receptors are structurally related typrosine kinase receptors selectively expressed on vascular endothelial cells and required for embryonic vascular development*
*TrkA/TrkB/TrkC*	*Tropomyosin-receptor-kinase receptor (Trk) family and their ligands, neurotrophins, control synaptic strength and plasticity in the mammalian nervous system*
**VEGF R1/Flt-1**	Receptor tyrosine kinase which plays a critical role in angiogenesis and is activated by VEGF-A, VEGF-B, and placental growth factor.
**VEGF R2/KDR**	Tyrosine-protein kinase that acts as a cell-surface receptor for VEGF-A, VEGF-C and VEGF-D.
**VEGF R3/Flt-4**	VEGF R3 (Flt-4), similarly to VEGF R1 (Flt-1) and VEGF R2 (KDR/Flk-1), belongs to the class III subfamily of receptor tyrosine kinases (RTKs). All three receptors contain seven immunoglobulin-like repeats in their extracellular domains and kinase insert domains in their intracellular regions. The expression of VEGF R1, 2, and 3 is almost exclusively restricted to the endothelial cells. These receptors are likely to play essential roles in vasculogenesis and angiogenesis.

In bold are reported Receptor Tyrosine Kinases with evidence of circadian rhythmicity of expression; see text for references

### GFR-dependent signaling and circadian pathways in the context of carcinogenesis

Cancer onset and progression is fueled by complex molecular mechanisms involving genetic and epigenetic changes at the level of driver oncogenes and/or controller tumor suppressor genes leading to deregulated activation of key signaling pathways, many of which are controlled by the biological clock and show circadian patterns of activation, for instance PI3K/AKT/mTOR, MAPK and JAK/STAT signaling pathways, crucially involved in the regulation of cell growth/survival, proliferation and differentiation.

### PI3K/AKT and mTOR signaling pathways and the biological clock

The PI3K/AKT pathway operates downstream of EGFR and human epidermal growth factor receptor (HER)2 controlling essential cell processes comprising growth, proliferation, survival, and motility/migration. Deranged PI3K/AKT signaling pathway activation upkeeps cancer cell proliferation and survival, favoring metastatic spread and chemoresistance [35]. On its side, AKT serine/threonine kinase comprises various domains: (i) N-terminal pleckstrin homology (PH) domain, a sequence of 100 amino acids interacting with phosphoinositides produced by the lipid kinase phosphoinositide 3-kinase (PI3K), AKT (ii) kinase domain, located at the heart of the molecule and similar to other Ser/Thr protein kinases of the AGC kinase group, counting the cyclic-nucleotide-dependent family (PKA and PKG), the protein kinase C family, the β-adrenergic receptor kinase (βARK), the ribosomal S6 family among others; (iii) C-terminal regulatory domain, a sequence of approximately 40 amino acids including the hydrophobic motif, whose phosphorylation initiates enzymatic activity [35]. Upon activation, AKT limits cell apoptosis and growth promoted by TGF-β and C/EBPα, respectively, and may turn on β-catenin downstream signaling. PI3K/AKT and TGF-β/SMAD signaling pathways interplay to control several cell processes, counting proliferation, apoptosis, and migration: TGF-β/SMAD signaling exerts cytostatic activity in premalignant states opposing pro-cell survival effect exerted by growth-factor-mediated PI3K/AKT activation, so that the two pathways might oppose each other in balancing cell survival [36]. In advanced cancers the anti-proliferative action of TGF-β is jammed and both TGF-β receptor-dependent and PI3K/AKT pathways exert pro-oncogenic effects through complex signal integration and obligated cooperation to support cancer development and progression [36]. The biological clock is synchronized by external and internal stimuli to cope with modifications in the environment or in the internal milieu and PI3K-dependent intracellular-signaling pathway was shown able to play a role in the fine-tuning of the molecular clockwork targeting BMAL1 and CLOCK and altering circadian DBP gene expression. PI3K knockdown by pharmacological inhibitors or shRNA-mediated silencing hindered DBP mRNA upregulation in NIH-3T3 cells (fibroblast cell line isolated from NIH/Swiss mouse embryo) synchronized with serum shock (treatment of cultured cells with high concentrations of serum) [37]. Furthermore, PI3K inhibition blocked BMAL1 and CLOCK heterodimerization, considerably decreased DBP gene promoter activity and diminished BMAL1/CLOCK heterodimer recruitment to the E-box DNA response element in the DBP promoter, with consequent transcriptional modulation of this important first order clock controlled gene [37]. The interplay of PI3K and circadian pathways was evaluated in the neural framework of cellular survival and a modulatory effect of BMAL1 protein together with PI3K/AKT signaling cascade regulation was shown, in addition to a supporting effect of melatonin on BMAL1 expression. BMAL1 and melatonin increased cellular survival after oxygen glucose deprivation with concurrent phosphorylation of AKT, ERK-1/2, PDK1, mTOR, PTEN, GSK-3αβ, and p70S6K, while PI3K/AKT inhibition diminished BMAL1 expression, suggesting that melatonin modulates BMAL1 expression through PI3K/AKT signaling and BMAL1 hampers cell death triggering survival kinases [38]. The PI3K/AKT pathway cooperatively interplays with the serine-threonine kinase mammalian target of rapamycin (mTOR), importantly involved in mRNA translation and protein synthesis [39, 40]. Remarkably, the circadian clock circuitry and mTOR signaling pathway interact at many levels and mTOR cascade rhythmic activation is driven by the biological clock in the SCN in mice [41]. Monitoring of the mTOR activity marker phosphorylated S6 ribosomal protein (pS6) evidenced maximal activity of the mTOR pathway during the subjective day and minimal activity during the late subjective night [41]. The circadian protein Per2 limits mTORC1 complex activity binding specifically and conjointly tuberous sclerosis complex 1/2 (TSC1/2), Raptor, and mTOR. On the other hand, PER2 expression is set off by the glucagon-Creb/Crtc2 signaling, with consequent mTORC1 suppression and PER2 protein absence substantially augments protein synthesis and cell proliferation [42]. Interestingly, regarding the cross-talk between PI3K/AKT pathway and circadian pathway in the context of carcinogenesis, an important role for this interplay was evidenced in cancer of the ovary, causing the highest mortality rate among gynecological tumors. In an in vitro model of ovarian cancer loss of PER2 elicited PI3K/AKT pathway activation and induced epithelial-mesenchymal transition, apoptosis inhibition, increase of drug efflux resulting in cisplatin resistance and inflammation [43]. In an animal model of renal carcinoma mTOR protein levels changed with circadian pattern and showed higher levels in tumor mass during the dark phase and mTOR-signaling pathway showed circadian activity rhythm driven by FBXW7-related protein degradation via the first order clock controlled gene DBP. Efficacy of antitumor therapy in renal cancer-bearing mice was higher if everolimus was administered when mTOR activity, and enticingly when sensitivity of renal tumors to everolimus, were higher [44]. Similarly, probing of an animal model of human lung cancer (xenograft of HCC827, an epithelial cell isolated from the lung adenocarcinoma of a white, 39-year-old female patient) and dosing time-dependent anti-tumor activity of erlotinib was assessed. Higher efficacy with greater tumor growth inhibition was observed when erlotinib was administered in the early light phase, coincident with amplified activity of EGFR and downstream PI3K/AKT and ERK/MAPK molecular cascades, suggesting evaluability of time-dependent optimized dosing schedule of this drug to obtain greater anti-tumor effects segregating effectiveness from dose-limiting toxicity with chronotherapy [45].

### MAP kinase signaling pathways and the Biological clock

The mitogen-activated protein kinase (MAPK) pathways, composed of a canonical three-tier hierarchy of serine/threonine kinases, are among the most highly conserved signaling pathways in eukaryotes and are activated by extracellular growth factors that bind to RTKs, operating in the cell *via* the RAS-RAF-MEK-ERK signaling cascade to transduce extracellular signals/stressors and trigger mitogenic responses through different transcriptional events in relation to the tissue type and the initiating stimulus [46, 47]. In mammalian cells, MAPK pathways encompass three principal families: extracellular-signal-regulated kinases (ERKs), Jun amino-terminal kinases (JNKs), and stress-activated protein kinases (p38/SAPKs), which are openly interconnected with the circadian pathways. Three successively triggered protein kinases activate these signaling pathways to start out cell growth, proliferation, differentiation, cell survival or apoptosis. These growth factor-mediated cell responses elicit transcription of 200–500 protein coding genes handling essential cell processes and are accompanied by tissue biological functions, such as development and inflammation, with eventually mutations in a number of oncogenes [46, 47]. In a wide range of neoplastic diseases, dysregulated activation of MAPK signaling occurs and is hastened via numerous mechanisms, including genetic mutations and/or anomalous expression of extracellular RTKs, setting off relentless and/or disproportionate receptor activity and signal transduction in the absence of appropriate ligand binding. This signaling derangement elicits augmented or uncontrolled cell proliferation and atypical cell survival [46, 47]. Circadian pathways interact in different ways with MAPK-dependent intracellular signaling and collaborate to accomplish important cell processes that when altered are entailed in carcinogenesis, mainly in tumor suppression [48]. In various eukaryotic organisms, phosphorylation of circadian proteins by each of the three MAPK subfamilies is definitely recognized and a role of MAPKs signal-transduction pathways in circadian input and output pathways is emerging [48]. In a time-of-day dependent manner the biological clock controls at the transcriptional level the expression of downstream tissue-specific genes enriching the MAPK pathway, with noticeable adjustment of different signaling events impacting biological responses [48]. On the other hand, MAPK family plays various important roles in the circadian clock circuitry and can impact the ticking of the biological clock permitting intracellular integration of external inputs to reset the phase of clock gene expression and sequentially the phase of rhythmic cell processes driven by the biological clock [49–51]. The role played by MAPK in biological timekeeping is crucial and prevails among the biochemical mechanisms that prop up entrainment of the circadian clock circuitry to environmental cues and in the mammalian SCN MAPK-dependent signal transduction entrain the circadian clock circuitry to diurnal cyclical environmental cues [49–51]. The various components hard-wiring MAPK signaling likewise cooperate genetically and/or at the molecular level with the cogs of the molecular clockwork. Importantly, in numerous model organisms and diverse tissue types MAPK pathway activation shows circadian patterns, affording coordinated and time-qualified transcription of tissue-specific target genes. MAPK signaling and circadian pathways impact analogous cell processes and biological phenomena and failing or changes in one or the other pathway are implicated in numerous analogous human diseases [52–54].

### JAK/STAT signaling pathway and the Biological clock

In humans, the Janus Kinase/Signal Transducer and Activator of Transcription (JAK/STAT) pathway is one of the key pathways involved in numerous biological processes, counting regulation of cell cycle, proliferation, and survival, among which intestinal stem cell and epithelial cell proliferation [55]. JAKs are actuated through cytokine/growth factors stimulation and phosphorylate STATs that dimerize and translocate into the nucleus where activate or suppress transcription of specific genes. Precisely, upon cytokines or growth factors binding, receptors coupled with JAKs go through conformational modification with ensuing JAKs activation, cross-phosphorylation and subsequent tyrosine phosphorylation at the cytoplasmic domains of the receptors [55]. This phenomenon leads to recruitment, phosphorylation and conformational change of STATs and phosphorylated STATs translocate into the nucleus where trigger transcription of target genes [55]. Suppressor of cytokine signaling (SOCS) 3 impedes STAT phosphorylation through suppression of JAKs activation [55]. JAK/STAT pathway shows circadian rhythmicity of activation with time-of-day dependent changes in regenerative responses. Tissue homeostasis preservation is an essential process, particularly in tissues with great regenerative capacity linked to high cell turnover, such as the intestine, and JAK/STAT signaling is crucially entailed in intestinal regenerative response. In case of cell damage the JAK/STAT pathway activates and elicits intestinal stem cells transition to rapid proliferation, increase of cell division and enteroblasts differentiation to rapidly substitute the damaged cells. Molecular clockworks endowed in intestinal stem cells drive division and fate during tissue regeneration and in turn the JAK/STAT pathway was shown able to impact circadian behavioral rhythms [56, 57]. JAK/STAT3-dependent signaling is considered as a key oncogenic pathway and its anomalous activation plays a role in onset and progression of various cancers. In ovarian cancer, suppression of the JAK/STAT3 signal significantly decreases tumor progression and metastatic spread and interestingly the molecular clockwork cog REV-ERBα, encoded by *NR1D1* gene, is down-regulated but is capable to hinder JAK/STAT3 pathway activity through SOCS3 expression up-regulation [58]. Hence, *NR1D1* over-expressing xenografts developed more slowly respect to controls and SOCS3 silencing obliterated the inhibitory activity of *NR1D1* over-expression on JAK/STAT3 signaling pathway and ovarian cancer progression [58]. Accordingly, analysis of data from GEPIA (Gene Expression Profiling Interactive Analysis), a web-based tool to deliver fast and customizable functionalities based on TCGA and GTEx data, showed that SOCS3 and *NR1D1* expression in ovarian cancer were positively correlated [58, 59].

### Circadian patterns of growth factor receptors ligands

#### Epidermal growth factors

The function of the circadian clock circuitry and cell/tissue response to mitogenic stimuli are strictly connected: the biological clock drives rhythmic fluctuations in GFR-dependent signaling that in turn modulates ticking of the molecular clockwork. A study performed in murine neural stem cells showed that cell cycle progression was synchronized with circadian rhythmicity upon epidermal growth factor (EGF) stimulation and in accordance with *PER2* gene expression pattern [60]. EGF challenge transitorily elicited the expression of numerous core clock genes, counting *PER1* and *PER2*, in addition to clock controlled genes such as *DEC1*, *E4BP4*, and *NOCT* (encoding Nocturnin), while pretreatment with a MEK1/2 inhibitor considerably hindered the short-term transcription of *PER2*, *DEC1*, and *NOCT* triggered by EGF, suggesting a specific effect of EGF on the molecular clockwork [60]. EGF-receptor signaling is regulated with circadian pattern by glucocorticoids and signal transduction dependent on nuclear receptors for steroid hormones (GR) and RTKs is necessary for cell and tissue homeostasis, as well. EGFR-dependent signaling is hampered by glucocorticoids through a time-of-day dependent mechanism: hindrance of activating feedback loops and concurrent eliciting of inhibiting feedback loops, customarily maneuvering the molecular cascade downstream of EGFR [61]. The circadian negative control of EGFR signaling by high glucocorticoid levels is operated during the active phase (at night in rodents), whereas during the resting phase (day-time in rodents) EGFR signaling is enhanced [61]. Accordingly, studies performed in animal models with EGFR-driven cancers corroborated diurnal opposing cycling of feedback loops related to EGFR-dependent signaling and showed better response in animals treated with a specific kinase inhibitor during the resting phase of the day, corresponding to lower glucocorticoid levels [61]. Besides, analysis of the METABRIC data set (integrating genomic and transcriptomic profiles of 2000 clinically annotated primary breast cancer specimens from United Kingdom and Canada tumor banks) with classification of patients into two equal size groups according to high and low GR transcript levels or into three groups according to tumor stage and GR abundance, showed better prognosis and longer patient survival time in breast cancer patients with high GR, low MAPK activity, whereas low GR expression was seemingly associated with higher ERK activation and predicted poorer prognosis [61, 62]. On the whole, the newly discovered regulation of EGFR-dependent signaling by glucocorticoids in the context of breast cancer, the most commonly occurring malignant neoplastic disease in women and the most frequent cancer overall, operated through the circadian interplay between EGF/RTKs-dependent signaling and glucocorticoids/GR as well as the greater efficacy of EGFR-inhibiting drugs and tyrosine kinase inhibitors administered in breast cancer animal models during the active phase of the day suggest evaluability of chronomodulated therapy schedules in EGFR-driven breast cancer patients [61].

#### Transforming growth factors

Studies performed in a mouse model suggested that EGFR signaling is involved in the circadian control of behavioral cycles. Transforming growth factor (TGF)-α was expressed rhythmically in the SCN, bound to EGF receptors on neurons in the hypothalamic sub-paraventricular zone and limited locomotor activity [63]. On the other hand, TGF-β and the phosphorylated form of SMAD3 protein are expressed under basal conditions in a time-of-day and age-dependent way in the SCN and para-ventricular nuclei (PVN) [64]. Accordingly, an interplay was shown at the molecular and behavioral level between the TGF-β signaling and the circadian pathway, suggesting that TGF-β signaling plays a significant role for proper circadian clock circuitry function [65]. TGF-β plays a role in coupling peripheral oscillators through paracrine pathways and canonical TGF-β signaling to fine-tune oscillation phase of molecular clockworks by means of core-clock genes transcriptional regulation. Intercellular peripheral coupling involves a molecular mechanism initiated by TGF-β secretion by peripheral oscillators and extracellular activation by the interaction with integrins, binding to specific transmembrane receptors, SMAD2 and SMAD4 proteins expression, complexation and nuclear translocation with transcriptional activation of TGF-β target genes [66]. In a murine model of progressive kidney disease, TGF-β mRNA expression showed circadian oscillation and was regulated by binding of CLOCK: BMAL1 heterodimers to E-box site near the 5′ end of the coding gene [67] Besides, in a murine lung pathology model TGF-β1 was found able to uphold BMAL1transcription and in turn BMAL1 was necessary for TGF-β1-induced signaling transduction [68]. Dysregulation of RSTK signaling and TGFβ/SMAD4 pathway in particular plays a role in human neoplastic diseases comprising gastrointestinal and pancreatic malignancies. A study performed using an in vitro model of human pancreatic ductal adenocarcinoma (PDA) suggested that TGFβ/SMAD4 pathway, commonly found mutated in PDA, is controlled by the molecular clockwork, with TGFβ1, SMAD3, SMAD4, and SMAD7 expression showing oscillation with circadian pattern [69]. TGFβ/SMAD4 pathway derangement impacted metastatic phenotype and cell fate in control and SMAD4 knocked-out pancreatic cancer cells [69]. Besides, DEC1, DEC2, and CRY1 expression changed according to SMAD4 expression in the pancreatic cancer cell, suggesting that the interplay between TGFβ canonical signaling and circadian pathway may crucially impact PDA malignancy and patient survival [69].

#### Vascular endothelial growth factors

In mammals, angiogenesis defines the biological phenomenon through which new blood vessels sprout and develop from the existing vasculature. This bioprocess is indispensable for developmental angiogenesis in manifold tissues and organs in embryos ad tissue growth, but when deregulated supports cancer progression [70, 71]. Members of the vascular endothelial growth factor (VEGF) family include VEGF-A, -B, -C, -D, and placental growth factor (PlGF), which bind to three RTKs, VEGFR-1, VEGFR-2, and VEGFR-3 located on the plasma membrane of endothelial cells of blood or lymphatic vessels [70, 71]. VEGFR-2 represents the functional receptor that transduces angiogenic and vascular permeability signals in response to VEGF-triggered signaling. Vascular sprouting, remodeling and maturation are restrained by the Dll4-Notch signaling system [70, 71]. The biological clock drives the rhythmic expression of the gene encoding one of the main angiogenic factors, VEGF, the growth factor holding up blood vessel growth, through modulation of the inhibitory action on vascular sprouting exerted by NOTCH signaling [70, 71]. Studies performed in the zebrafish (Danio rerio) showed that VEGF expression levels oscillate with circadian periodicity and the mRNA expression level of the encoding gene is controlled by BMAL1, which targets the *VEGF* promoter for transcriptional activation and drives 24-hour rhythmic modulation of developmental angiogenesis, opposing the anti-angiogenic effects of PER2 [70, 71]. Neo-angiogenesis is crucial for tumor growth, with metastatic spread relying on neovascularization, and in cancer patients a valuable strategy for therapy aims at targeting pathological blood vessels in tumor tissues [70, 71].

VEGF expression is significantly amplified in malignant tumors and higher expression levels predict worst prognosis in numerous cancer types. Among different stimuli, hypoxia plays a key role in cancer-induced/associated neovascularization and in hypoxic tumor cells VEGF expression is rhythmically driven by the molecular clockwork [72]. Studies performed in animal models showed that VEGF mRNA levels increased significantly upon hypoxic challenge (prompting HIF production) in murine tumor cells (sarcoma 180, B16 melanoma, Lewis lung carcinoma cells) implanted in mice and oscillated with circadian pattern [72]. CRY1 and PER2 levels likewise oscillated with circadian rhythmicity in the implanted tumor cells and hampered hypoxia-induced VEGF transcription, signifying that the negative limb of the TTFL rhythmically prevents the hypoxia-dependent activation of VEGF transcription, driving 24-hour periodicity of mRNA expression oscillation [72]. Importantly, therapy with antiangiogenic agents prompted enhanced antitumor effects when administered in coincidence with the diurnal time-window corresponding to greater VEGF production, suggesting that time-qualified schedules in line with VEGF production could be a valuable strategy for administration of anti-angiogenic drugs at the best applicable time-of-day for the treatment of several solid tumors [72].

#### Platelet-derived growth factors

Platelet-derived growth factors (PDGFs) are homodimers or heterodimers of two distinct A and B polypeptide chains with 60% homology, encoded by two genes, PDGF A and B, and constitute a family comprising five isoforms: PDGF-AA, PDGF-AB, PDGF-BB, PDGF-C, and PDGF-D, operating through two RTKs, PDGF-Rα and PDGF-Rβ, located in plasma membranes of cell of mesenchymal origin [73] (Nakagawa H, 2008). The predominant in vivo PDGFs/PDGFRs interaction in the vascular system takes place between PDGF-BB and PDGFR-β. PDGFs were firstly discovered as platelets constituent released in the occasion of platelet activation and blood coagulation. PDGFs prompt growth, differentiation, survival and chemotaxis of target cells [73]. PDGF overexpression plays a role in malignant neoplastic diseases supporting tumor progression via autocrine stimulation of tumor cell growth, recruitment of tumor stroma fibroblast, and induction of tumor angiogenesis [74]. Nevertheless, the proliferation rhythm of cancer cells is often different respect to normal cells and DNA synthesis in sarcoma cells showed circadian rhythmicity different from that of normal cells. Pharmacologic inhibition of PDGF-RTKs related cascade significantly change DNA synthesis rhythm in sarcoma cells implanted in mice suggesting a modulatory role for enhanced PDGF receptor signaling, which is activated in cancer progression. Because the rhythmic patterns of clock gene expression in tumor cells did not differ significantly [76]. In addition, rhythmic changes in the tyrosine kinase activity of PDGF receptors in cancer cells apparently influence dosing time-dependent tumor growth inhibition efficacy of imatinib mesylate. Imatinib mesylate inhibits the function of various RTKs, including ABL, the BCR-ABL chimeric product, KIT, and PDGF receptors. Anti-tumor effect was greater when imatinib mesylate was administered in the early light phase, when PDGF receptor activity was enhanced, and drug efficacy was found related with drug-induced inhibition of PDGF receptor activity [75].

#### Fibroblast growth factors

Fibroblast growth factor (FGFs) family members signal as ligand­dependent dimers through RTKs and intermolecular transphosphorylation of the tyrosine kinase domains and intracellular tail recruit SH2 (Src homology-2) or PTBs (phosphotyrosine-binding domains) of adaptors docking proteins resulting in a cascade of phosphorylation events-dependent signaling, mainly RAS/MAP kinase pathway and to a lesser extent PI3K/AKT pathway and PLCγ pathway to regulate cell proliferation, survival, migration, and differentiation [76, 77]. The FGF family includes 23 members, even though only 18 FGFR ligands exist and four family members do not bind with FGFR as FGF homologous factors (FGF11, FGF12, FGF13, and FGF14) and no human FGF15 gene exist, while the gene orthologous to mouse FGF15 is FGF19 [76, 77]. Human FGFs comprise 22 mitogenic and non mitogenic members with a molecular mass ranging from 17 to 34 kDa and sharing 13–71% amino acid identity (FGF1, FGF2, FGF3 (INT2), FGF4, FGF5, FGF6, FGF7 (KGF), FGF8 (AIGF), FGF9, FGF10, FGF11, FGF12, FGF13, FGF14, FGF16, FGF17, FGF18, FGF19, FGF20, FGF21, FGF22, and FGF23) [76, 77]. The circadian clock circuitry drives 24-hour rhythmic fluctuation of FGFs in physiological and pathological conditions [78, 79]. Deranged expression of some FGFs and their receptors can play tumor specific and opposing oncogenic or tumor suppressive roles in carcinogenesis. Interestingly, in breast cancer patients the circulating levels of basic fibroblast growth factor (bFGF, FGF-2), epidermal growth factor (EGF) and insulin-like growth factor-1 (IGF-1) showed circadian patterns of fluctuation [80]. Furthermore, a study performed using chronic jet lag in a cancer cell model (U2OS, human osteosarcoma cells, expressing a Period2 promoter-driven destabilized luciferase reporter, consecutively 8-hour advanced with dexamethasone treatment cycles every 2 days) and in a cancer animal model (artificially jet-lagged mice inoculated with B16/F10 murine melanoma cells or treated with methylcholanthrene) showed that circadian dysregulation upholds CDK4/6-dependent phosphorylation of retinoblastoma protein (pRB) with ensuing pro-proliferative signaling events and promotes cell cycle progression with G1/S phase transition [81]. Transcriptomic analysis evidenced significant upregulation of genes encoding crucial ligands and receptors of FGF/FGFR in addition to EGF/EGFR and VEGF/VEGFR signaling pathways, suggesting valuable targets for time-of-day qualified chemotherapy schedules [81, 82].

## Conclusion

Disruption of the circadian clock circuitry by genetic and environmental factors enhances susceptibility to multiple cancer types and in 2007 the WHO International Agency for Research on Cancer (IARC) Working Group classified circadian rhythm disruption as plausible (type 2 A) carcinogenic. Numerous and diverse events that regulate cell proliferation, growth, differentiation and survival are managed by molecular pathways prompted by phosphorylation of tyrosine and serine-threonine residues of cell-surface and membrane-bound enzyme-linked receptors upon specific ligand binding. Molecular clockworks ticking in every normal and cancer cell type drive rhythmic oscillations of GFR-dependent signaling impacting the bio-processes that are critical for carcinogenesis when deregulated. On the other hand, the rhythmic patterns of fluctuation of these crucial GFR-dependent signaling pathways represent potential and valuable targets for cancer chrono-therapeutic approaches, timing chemotherapy with the peaks-and-troughs in the activity of these crucial molecular cascades and the 24-hour rhythms of efficacy and/or tolerability of anti-tumor drugs according to dosing time. Our fundamental aim and the principal scope of this review article was to highlight an undeniably crucial and original issue to be considered when exploring the pathogenic mechanisms supporting cancer onset and progression, i.e. the control carried out by the biological clock on GFR-dependent intracellular cascades, which consequently are featured by rhythmic activation and signaling patterns. This groundbreacking whelm of information opens up scenarios that are at present not sufficiently explored in basic and clinical cancer research as well as not properly exploited in targeted therapeutic strategies for patient care in clinical practice.

## Data Availability

No datasets were generated or analysed during the current study.
